# Long-term locked knee ankle foot orthosis use: A perspective overview of iatrogenic biomechanical and physiological perils

**DOI:** 10.3389/fresc.2023.1138792

**Published:** 2023-05-04

**Authors:** Kamiar Ghoseiri, Audrey Zucker-Levin

**Affiliations:** School of Rehabilitation Science, College of Medicine, University of Saskatchewan, Saskatoon, SK, Canada

**Keywords:** orthotic devices, locked knee joint, long-term adverse effects, knee ankle foot orthosis, biomechanics, physiological perils

## Abstract

A knee ankle foot orthosis (KAFO) may be prescribed to the person with severe neuromusculoskeletal impairment of the lower limb to promote walking stability. The locked knee ankle foot orthosis (L-KAFO) is among the KAFO's routinely prescribed; however, long-term use of the L-KAFO is associated with musculoskeletal (arthrogenic and myogenic) and integumentary changes, and gait asymmetry with increased energy expenditure. Consequently, the risk of developing low back pain, osteoarthritis of the lower limbs and spinal joints, skin dermatitis, and ulceration increases, all of which impact quality of life. This article synthesizes the iatrogenic biomechanical and physiological perils of long-term L-KAFO use. It promotes using recent advances in rehabilitation engineering to improve daily activities and independence for proper patient groups.

## 1. Introduction

The knee ankle foot orthosis (KAFO) is a wearable passive or active assistive device custom-made to conform to the lower limb of patients with instability or severe motor impairment. The KAFO is prescribed to increase functional abilities, independence, and social participation, which impacts the quality of life (1–3). The KAFO is designed to stabilize, unload, or immobilize the knee and ankle joints, protect the limb from injury, improve limb alignment, assist with motion, and/or generate torque at the knee (4). The knee joint mechanism is the critical component affecting KAFO functionality. Knee mechanisms are designed to be free, locked, or stance-controlled depending on muscular strength and joint kinematics. The free knee mechanism allows the knee to flex and extend during both the stance and swing phases of gait while providing mediolateral stability to the knee. The locked knee mechanism (e.g., drop-lock, bail-lock, Swiss-lock) maintains the knee in extension throughout the entire gait cycle imposing an energy-inefficient long-legged gait pattern (circumduction, vaulting, or hip hiking) on the wearer and must be released mechanically or manually for sitting. The stance control KAFO (SC-KAFO), introduced in the 2000's, evolved the knee joint mechanism by stabilizing the knee in the stance phase while permitting free knee motion in the swing phase. This allows a more aesthetically pleasant and symmetric gait pattern with less energy expenditure and higher walking speed when compared to walking with a locked-KAFO (L-KAFO) (5, 6).

Although the benefits of swing phase knee motion provided by SC-KAFO are evident, the L-KAFO remains the most prevalent orthotic intervention for patient in need of a KAFO (7–9). L-KAFO must be prescribed in the presence of hip or knee flexion contractures >10 degrees, leg length discrepancy >15 cm, genu varum/valgum >10 degrees, body weight >125 kg, hip musculature strength <grade 3 (fair), the need for ischial weight-bearing, cognitive and psychological disorders, and those with bilateral motor impairment or flail lower limbs (8, 9). However, most patients devoid of these limitations are not prescribed SC-KAFO primarily due to cost and the need for training, predisposing the wearer to iatrogenic consequences associated with long-term use. This article synthesizes the potential perils of long-term L-KAFO use.

## 2. Abnormal gait patterns

Restricted knee flexion created by a locked knee joint leads to gait asymmetry. Insufficient knee flexion during swing impairs toe clearance forcing the wearer to adopt a long-legged gait compensation (circumduction, vaulting or hip hiking) which introduces the danger of tripping. Conversely, insufficient knee flexion at initial contact and during pre-swing leads to increased energy expenditure from the inability to smoothly progress the body's center of gravity (COG) over the supporting foot. Further, the long-legged gait pattern is associated with pain and deformity in other body parts (5, 6). For instance, during the transition from loading response to midstance, fixing the knee joint in extension promotes hyperextension of the hip and sway back posture of the trunk with increased lumbar lordosis and thoracic kyphosis. Therefore, the hip flexor muscles must exert more force to generate higher moments to maintain an upright posture which may contribute to anterior hip pain and fatigue (10).

In normal walking, energy is conserved by minimizing the excursion of the COG. For example, slight knee flexion at initial contact and terminal stance decreases the vertical displacement of the COG; however, this strategy is eliminated with L-KAFO use leading to increased energy expenditure as identified by the finding that able-bodied people walking on a level surface with an immobilized knee expend 20%–22.7% more energy than when walking freely (11, 12). Energy expenditure is heightened for people in need of a KAFO as the physical limitations for which they need the KAFO, such as quadriceps weakness, are now compounded by the imposed weight and gait deviation of the KAFO (13). The weight of a regular KAFO, approximately 5 lb (2.27 kg) (1), increases stance phase duration, and decreases walking velocity, cadence, and peak hip flexion (14). Imposed long-legged gait deviation, including ipsilateral leg circumduction, hip hiking (hip elevation), and trunk lateral sway occurring during the swing phase (1), while contralateral vaulting (ankle plantarflexion) at terminal stance also contributes to increased energy expenditure (15). Interventions to diminish energy expenditure, including the use of a contralateral shoe lift to aid with ipsilateral toe clearance, have improved oxygen consumption rate and energy expenditure demands when compared to KAFO use alone, but remain higher than unaided walking (12). Walking aids, such as a cane, crutch, and walker, are commonly used in addition to the KAFO to provide supplemental support and safety and have been shown to contribute to increased energy expenditure (16).

## 3. Shock absorption

During gait, specifically at initial contact, the body weight abruptly transfers to the stance limb. The ground reaction force (GRF) at initial contact needs to be attenuated to minimize trauma to the lower limb and spinal joints. Viscoelastic properties of the soft tissues and active kinematic change in the lower limb and spine joints are the main mechanisms for shock absorption (17). Subtalar pronation, ankle plantarflexion, knee and hip flexion, and pelvic obliquity control the descent of body mass, which in turn diminishes the GRF at initial contact (18). When walking with an L-KAFO, the capacity of lower limb shock absorption decreases. Further impairment presents when the ankle joint of the KAFO is also locked to give more support for the limb. The inability to dampen the GRF adequately at initial contact is associated with low back pain, and osteoarthritis of the lower limb and spinal joints due to gradual cartilage degeneration (17, 18).

## 4. Integumentary concerns

Pistoning, the vertical displacement of the limb inside the orthosis, caused by KAFO weight, inadequate suspension, misalignment of the uprights and shells, mismatch of the anatomical knee joint to the mechanical knee joint, and load-bearing of the limb inside the KAFO causes excessive shear forces at the skin-orthosis interface (19, 20).

The uniaxial mechanical knee hinge of the L-KAFO cannot adapt to the instantaneous center of rotation of the anatomical knee joint, creating torsional moments when the locking mechanism is released, i.e., during sitting. These torsional moments lead to shear stress over the skin and may cause friction blisters.

In addition to shear stress, the large plastic thigh and calf shells of a KAFO may lead to heat buildup and skin perspiration. Consequently, skin maceration caused by moisture is associated with a change in the skin's constant of friction and stiffness. These changes impact the mechanics at the skin-orthosis interface and lead to skin dermatitis and ulceration (21). Moreover, the hot and moist environment inside KAFO provides an ideal environment for bacterial growth, contributing to skin infection and unpleasant odor.

## 5. Physiological concerns

A KAFO with a locked knee mechanism causes joint immobilization. Immobilization leads to arthrogenic (i.e., bone, cartilage, synovial membrane, joint capsule, and ligaments) and myogenic (i.e., muscle, tendon) changes in synovial joints. Long-term immobilization leads to the shortening of the synovial capsule, adhesion of the synovial membrane, decreases synovial fluid production and its diffusion into the joint cavity, which consequently causes a decrease in lubrication of the joint surfaces, increase in the coefficient of friction, and increase in stiffness of the joint (22). Long-term immobilization and partial unloading of a joint lead to cartilage softening, reducing cartilage proteoglycan content and decreasing cartilage thickness (23). For instance, in the knee joint, the largest synovial joint of the human body, long-term immobilization (e.g., 8 weeks or more) impairs the arthrokinematics and kinetic friction-related characteristics (22). Immobilization leads to stress deprivation and an increase in the cross-sectional area of the ligament (24). It also induces a change in the stress-strain curve of the ligament mainly by reducing the modulus of elasticity (24).

Although some specific KAFOs are designed with ischial/quadrilateral weight-bearing thigh brims to unload the lower limb, e.g., those needed for non-union fractures of the lower limb, even a regular KAFO with a locked knee mechanism has some unloading effect (25). The uprights and shells of the KAFO partially share (range, 30%–83%) the load that transfers through the lower limb to the ground (19). Therefore, with long-term use, less mechanical stress passes through the lower limb segments, which consequently affects the biomechanics of the lower limb tissues (26). Reduced mechanical stress on lower limb bones leads to disuse osteoporosis (27), localized reduction of bone mineral density, content, and strength (26).

In addition to osseous changes, myogenic changes including muscle atrophy, shortness of the muscle, decreased cross-sectional area of muscle fibers, increased oxidative stress (28) and inflammatory response of the muscle tissue may occur with L-KAFO use (29). Even short-term immobilization is associated with changes in muscle force production, changes in corticospinal excitability, and a reduction in the amplitude of evoked motor potentials (30).

Static orthoses that limit joint motion may cause muscle disuse (31). It has been shown that immediately after using a static AFO, the electromyography (EMG) activity of the tibialis anterior muscle is reduced by 20% in healthy people and 7% in people with unilateral paretic drop foot (31). Muscle activity and strength of the lower limbs could differ among patients, pending the clinical impacts of each pathology and its severity. Therefore, using a KAFO can influence the remaining muscle activity of each lower limb differently. There is limited evidence on the impact of orthoses on muscle activity of the lower limbs. However, it could be theorized that the muscle activity level in KAFO users corresponds to the compensatory walking patterns, placement of the GRF with respect to the lower limb joints, and the impact of increasing the weight of the orthotic lower limb. For example, contralateral gluteus medius, internal oblique, and external oblique abdominal muscle activity increases when a weight corresponding to 1% or 2% of the body mass is used above the malleoli (32).

## 6. Future directions

The iatrogenic effects of long-term L-KAFO use should be closely examined to identify who is at the most risk. Customization of the KAFO and selection of proper fabrication materials with respect to stiffness and weight must also be considered. The stiffness of the KAFO structure, i.e., uprights and size of the shells, tuning the knee and ankle joints with respect to the GRF must be customized for each patient. In addition, exercise and muscle stretching are recommended for L-KAFO users to counter knee immobilization effects. Techniques to increase muscle activity, including employing advanced orthotic knee joint technology and/or haptic subthreshold sensory stimulation, which may increase lower limb muscle activity and minimize disuse atrophy, are recommended (33–35). Finally, improving health service policies and insurance coverage may allow deploying advanced rehabilitation engineering innovations for people who meet SC-KAFO criteria to experience less iatrogenic complications, more independence in daily activities, and improved quality of life.

## 7. Conclusion

While an L-KAFO has many functional, biomechanical, physiological, and psychosocial short-term and long-term benefits for standing and walking of a patient, its long-term use is associated with negative consequences. Using L-KAFO must be justified for each patient uniquely based on the remaining muscular activity level, and orthotic support required. Research on techniques and technologies to deal with the iatrogenic biomechanical and physiological perils of L-KAFO long-term use warrants further investigation as well as to determine their impact on quality of life.
